# Resequencing the Genome of *Malassezia restricta* Strain KCTC 27527

**DOI:** 10.1128/MRA.00213-19

**Published:** 2019-04-18

**Authors:** Yong-Joon Cho, Minji Park, Won Hee Jung

**Affiliations:** a Korea Polar Research Institute, Incheon, South Korea; b Department of Systems Biotechnology, Chung-Ang University, Anseong, South Korea; University of California, Riverside

## Abstract

The draft genome sequence of *Malassezia restricta* KCTC 27527, a clinical isolate from a patient with dandruff, was previously reported. Using the PacBio Sequel platform, we completed and reannotated the genome of *M. restricta* KCTC 27527 for a better understanding of the genome of this fungus.

## ANNOUNCEMENT

*Malassezia* species are recognized to be involved in skin diseases, including dandruff, seborrheic dermatitis, and atopic dermatitis. Among the 17 identified *Malassezia* species, *M. restricta* is the predominant species on human skin and is particularly associated with dandruff, as suggested by recent microbiome analyses (1–4).

We previously sequenced and analyzed the genome of *M. restricta* KCTC 27527, a clinical isolate from a patient with dandruff, in South Korea (5). The previous assembly generated 51 contigs that were assembled into 18 scaffolds containing 3,580 coding sequences (CDSs), representing an overall completeness of 89.7% with Core Eukaryotic Genes Mapping Approach (CEGMA) analysis (5, 6). To address the incompleteness of the previous genome sequencing, we resequenced and completed the genome of *M. restricta* KCTC 27527 in the current study.

*M. restricta* KCTC 27527 cells were grown in Leeming and Notman agar (LNA) medium (0.5% glucose, 1% peptone, 0.01% yeast extract, 0.8% bile salt, 0.1% glycerol, 0.05% glycerol monostearate, 0.05% Tween 60, 1.2% agar, 0.5% whole-fat cow milk, and 170 µg/ml chloramphenicol) at 34°C for 3 days (7), and genomic DNA was extracted and a SMRTbell library was prepared according to the manufacturer’s instructions (8). Genome sequencing was performed using P6-C4 chemistry on one cell of a PacBio Sequel platform (Pacific Biosciences). Raw reads were *de novo* assembled using the Canu v. 1.7 assembler with the parameter “genomeSize = 7.3m,” and the assembled contigs were polished with the Arrow consensus caller in PacBio SMRT Link v. 5.0.1 (9). Telomeric motifs in chromosomal ends and mitochondrial contigs were manually curated. Discrepancies between contigs from the previously reported assembly and assembly of PacBio Sequel reads were corrected through the analysis using the CodonCode Aligner software package (CodonCode Corporation). The first round of gene prediction was performed with BRAKER v. 2.1.0 with the parameter “minimum intron length = 20” (10). In this process, *de novo* assembly of existing transcriptome sequencing (RNA-Seq) data on the Gene Expression Omnibus (GEO) database (accession number GSE112036) was used to reflect the exon-intron structure. RNA-Seq raw reads were cleaned by Trimmomatic v. 0.36 and mapped using Hierarchical Indexing for Spliced Alignment of Transcripts (HISAT) v. 2.1.0 (11, 12). After the first round, the BRAKER config file was modified by setting the normal penalty parameter for introns to 0 and the bonus for RNA-Seq data to 1e + 100 to reflect only introns derived from actual data. The final gene prediction was performed using AUGUSTUS v. 3.2.3 with the parameter “min_intron_len = 15” and the hints and config files generated in the processes discussed above (13). We corrected the gene structure where the splicing site differed from the actual transcript with the RNA-Seq mapping results in the Integrative Genomics Viewer (IGV) genome browser. Genome annotation was carried out with the NCBI RefSeq database release 88, eggNOG v. 4.5, and the KEGG database (14–16). Mitochondrial gene prediction and annotation were performed using MITOS2 (17).

As a result of the resequencing, all gaps were filled and the assembly was completed. A total of 9 chromosomes and a mitochondrion of 7,330,907 bp (GC content, 55.79%) and 38,720 bp (GC content, 31.4%), respectively, were estimated with a read coverage of 38.8×. Further, 4,390 CDSs, 29 rRNAs, 74 tRNAs, and 9 noncoding RNAs (ncRNAs) were identified in the annotated assemblies of the chromosomes. The annotated mitochondrial genome contained 16 CDSs, 2 rRNAs, and 24 tRNAs. The genome statistics are summarized in Table 1. The new assembly contained an additional 810 and 3 predicted CDSs in the chromosomes and mitochondrion, respectively, compared to our previous assembly.

**TABLE 1:** Summary of genome statistics for *M. restricta* KCTC 27527

Genome statistic	Value
Genome size (bp)	7,330,907
No. of chromosomes	9
Chromosome size range (Mbp)	0.16–1.42
No. of genes (protein coding genes)	4,390
Mean gene length (bp)	1,492
Gene density (genes per Mb)	598.91
Genome GC content (%)	55.79
Mitochondrial genome size (bp)	38,720
Mitochondrial GC content (%)	31.4

### Data availability.

This whole-genome sequence has been deposited in GenBank under the accession numbers CP030251 to CP030260 from BioProject PRJNA477735.
